# Surgeons' preference sublobar resection for stage I NSCLC less than 3 cm

**DOI:** 10.1111/1759-7714.13336

**Published:** 2020-02-09

**Authors:** Chien‐Sheng Huang, Po‐Kuei Hsu, Chun‐Ku Chen, Yi‐Chen Yeh, Han‐Shui Hsu, Chun‐Che Shih, Biing‐Shiun Huang

**Affiliations:** ^1^ Division of Thoracic Surgery, Department of Surgery Taipei Veterans General Hospital Taipei Taiwan; ^2^ Institute of Clinical Medicine, School of Medicine Taipei Taiwan; ^3^ Department of Radiology Taipei Veterans General Hospital Taipei Taiwan; ^4^ Department of Pathology Taipei Veterans General Hospital Taipei Taiwan; ^5^ Division of Cardiovascular Surgery, Department of Surgery Taipei Veterans General Hospital Taipei Taiwan

**Keywords:** Early stage lung cancer, non‐small cell lung cancer, segmentectomy, sublobar resection, wedge resection

## Abstract

**Background:**

This study aimed to compare survival between standard lobectomy and surgeons' preference sublobar resection among patients with stage I non‐small cell lung cancer (NSCLC).

**Methods:**

Medical records of patients undergoing pulmonary resection between 2006 and 2016 were reviewed retrospectively. Differences in disease‐free survival (DFS) and DFS‐associated factors between patients receiving lobectomy and surgeons' preference sublobar resection were analyzed after 1‐1 propensity score‐matching (*n* = 119 per group).

**Results:**

In total, 1064 pathological stage I NSCLC patients were identified, including 816 (76.7%) who underwent lobectomy, 111 (10.4%) who underwent sublobar resection as a compromised procedure (medically unfit), and 137 (12.9%) who underwent surgeons' preference sublobar resection. Rates of five‐year DFS for patients undergoing lobectomy, medically unfit, and surgeons' preference sublobar resection were 88.7%, 71.0%, and 93.4%, respectively (*P* < 0.001). Multivariable Cox regression analysis demonstrated that radiological solid‐appearance (adjusted hazard [aHR] = 2.908, *P* = 0.003), PL2 invasion (aHR = 1.970, *P* = 0.024), and angiolymphatic invasion (aHR = 2.202, *P* = 0.005) were significantly associated with lower DFS after adjusting for surgeons' preference sublobar resection (aH = 1.031, *P* = 0.939). Subgroup analysis of all 403 solid‐dominant patients demonstrated equivalent five‐year DFS between surgeons' preference sublobar resection and lobectomy (87.7% and 84.1%, respectively, *P* = 0.721). Propensity‐matched analysis showed no differences in five‐year DFS in stage I NSCLC patients undergoing lobectomy or surgeons' preference sublobar resection (90.5% vs. 93.4% *P* = 0.510), and DFS for surgeons' preference sublobar resection remained an insignificant factor (aHR = 0.894, *P* = 0.834).

**Conclusions:**

Carefully selected patients who have undergone surgeons' preference sublobar resection have comparable outcomes to those receiving lobectomy for stage I NSCLC <3 cm.

**Key points:**

Significant findings of the study Intended sublobar resection has a good outcome. What this study adds Sublobar resection is applicable for stage I NSCLC <3 cm.

## Introduction

In recent years, more and more small, peripheral and indolent pulmonary nodules have been diagnosed due to the increasing availability and advocacy of screening programs using low‐dose chest computed tomography (CT). Surgical resection is still the gold standard for treating these early stage lung cancers. Although mature results from trials in North America (Cancer and Leukemia Group B 140503) and Japan (Japan Clinical Oncology Group 0802) of lobectomy versus sublobar resection will not be released until 2020, sublobar resection, as an alternative treatment for peripheral small pulmonary malignancies, has accumulated evidence as a parenchyma‐preserving surgical approach for early‐stage non‐small cell lung cancer (NSCLC).^1^, ^2^

Currently, retrospective studies from single‐institute or population‐based analysis demonstrate controversial outcomes regarding the extent of lung resection. The surgical results from the literature are difficult to interpret because patients undergo sublobar resections for diverse reasons. Excluding intentional purposes developed in recent years, the majority of patients who previously underwent sublobar resection were high‐risk patients limited by decreased cardiopulmonary function or the presence of significant comorbid disease receiving compromised procedures.

It is more appropriate to compare such clinically judged high‐risk patients, who have no choice but to undergo sublobar resection, with similar high‐risk patients who received other treatment modalities such as stereotactic body radiation therapy or radiofrequency ablation,^3^ rather than comparing them with those who received standard lobectomy. In addition, most such studies have analyzed small, peripheral or slow‐growing NSCLC, once called bronchoalveolar carcinoma but since renamed pre‐ or minimally invasive adenocarcinoma. Such cases have excellent surgical outcomes, even with a simple wedge resection or without an adequate free margin.^4^, ^5^, ^6^

For these reasons, the role of sublobar resection in stage I NSCLC with an intentional purpose, instead of being a compromised procedure or being used for pre‐ or minimally invasive adenocarcinoma is still undetermined. To clarify this issue, the present study aimed to evaluate the surgical outcomes of stage I NSCLC <3 cm between patients undergoing standard lobectomy and surgeons' preference sublobar resection.

## Methods

### Patient selection

The medical records of patients who underwent pulmonary resection for stage I NSCLC from January 2006 to December 2016 at Taipei Veterans General Hospital were reviewed. Clinical and demographic characteristics including age, sex, smoking history, pulmonary function, preoperative serum carcinoembryonic antigen (CEA) level (normal range: <6 ng/mL), histologic tumor type, tumor size, presence of lymphovascular invasion, presence of pleural invasion, and methods of surgical approach were recorded. Patients received lobectomy or sublobar resection according to the judgment of thoracic oncologic experts of the surgical risk and after discussion with the patient. Lobar resection was usually performed in all patients with a good performance status and adequate pulmonary function tests. The reasons patients underwent sublobar resections as compromised or intentional‐purpose procedures were recorded into the operative notes. The study protocol was approved by the hospital Institutional Review Board (2) of Taipei Veterans General Hospital and informed consent from patients was waived (approval no. 2019‐01‐036BC).

**Figure 1:**
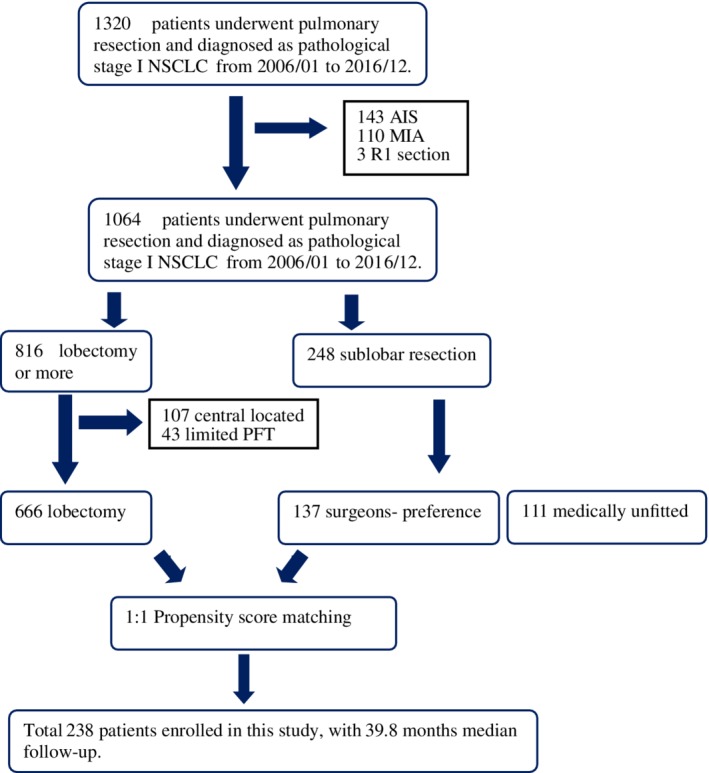
Flow diagram for the patient selection included in this study.

During the study period, 1320 patients underwent pulmonary resection for pathological stage I NSCLC. To compare the role of surgeons' preference sublobar resection with lobectomy, our exclusion criteria included: (i) Patients with pre‐ or minimally invasive adenocarcinoma (no tumor recurrence after either surgical approach in our hospital); (ii) patients with R1 section; (iii) patients with sublobar resection as a compromised procedure (medically unfit); (iv) patients with central‐type NSCLC, and (v) patients with limited pulmonary function tests who had undergone lobectomy. Finally, the data of 803 patients with stage I NSCLC was included for further analysis (Fig 1).

### Surgery

Mediastinal evaluation included mediastinoscopy, endobronchial ultrasound lymph node aspiration, intraoperative lymphadenectomy, or preoperative positron emission tomography (PET) scan. Patients either underwent radical mediastinal lymphadenectomy (the majority) or mediastinal node sampling according to the surgeon's preference. Adequate lymph node sampling was defined as removal of at least 15 lymph nodes and included three N2 stations.^7^

### Medically unfit versus surgeons' preference sublobar resection

The standard surgical resection of early stage lung cancer in our institute is lobectomy with lymph node dissection. The reasons why patients undergo a suboptimal surgical resection are recorded on the operative reports. According to the review of the operative reports of the reasons for a compromised surgical procedure instead of standard lobectomy, patients who underwent sublobar resections were defined as medically unfit. These patients were aged >80 years, had limited preoperative pulmonary function tests (either preoperatively forced expiratory volume in one second or diffusing capacity of the lungs for carbon monoxide <50%), had a second primary solid malignancy, or had other comorbidities defined as “high‐risk” in the literature.^8^ The surgical procedure for patients who underwent sublobar resections with intentional purpose was defined as surgeon's preference sublobar resection.

### Preoperative radiological evaluation

Radiologic findings of tumor were defined by thin‐section CT or involved multidimensional slicing and reconstruction into axial, coronal, and sagittal views. Tumor characteristics from the preoperative chest CT were read by two independent observers and the tumor size was reviewed in detail. In addition, all tumors were evaluated to estimate the extent of ground‐glass opacity (GGO) using the same thin‐section CT scan with a 2 mm collimation (GE Healthcare, Chicago, IL, USA). The lung was photographed with a window level of −500 to –700 H and a window depth of 1000 to 2000 H as the “lung window,” and a window level of 30 to 60 H and a window depth of 350 to 600 H as the “mediastinal window.” The consolidation tumor ratio (CTR) was defined as the ratio of the maximum size of consolidation to the maximum tumor size on thin‐section CT scan. Based on a CTR, a part‐solid tumor was defined as a tumor with both a focal nodular opacity and GGO (0 less than CTR ≤1.0), classed into two groups: GGO‐dominant (0 less than CTR 0.5) and solid‐dominant (0.5 less than CTR ≤1.0).^9^ The solid group (CTR = 1.0) was also evaluated separately as a variable in survival analysis.^10^

### Pathological examination

The pathologic stage was determined using the eighth TNM system for lung cancer.^11^ Visceral pleural invasion (VPI) was classified according to the proposal of the International Association for the Study of Lung Cancer (IASLC).^12^ VPI was first examined in tumor sections stained with hematoxylin and eosin. Elastic stains were performed in tumor sections when the status of VPI was indeterminate by hematoxylin and eosin stains.^13^ PL1 and PL2 indicate VPI and are a T2 descriptor. In the current study, pathologic stage PL0 was defined as without VPI, whereas pathologic stages PL1 and PL2 were defined as with VPI. Angiolymphatic invasion was defined as the presence of either vascular invasion or lymphatic permeation. For intentional‐purpose sublobar resection, surgical margins were defined as appropriate if they were more than 2 cm or at least equal to the diameter of the tumor according to the pathology report.

### Follow‐up

Operative mortality included death from all causes occurring within 30 days of surgery or after 30 days but during the same hospitalization period. Postoperative surveillance was scheduled every three months for the first two years, every six months for the third to fifth years, and annually thereafter. Chest CT scan was performed every six months for two years, then annually. Locoregional recurrence was defined as tumor recurrence in a contiguous anatomic site, including the ipsilateral hemithorax and mediastinum, after surgical resection. Distant recurrence was defined as tumor recurrence in the contralateral lung or outside the hemithorax and mediastinum after surgical resection. Recurrences were confirmed by tissue biopsy or clinically determined by the multidisciplinary lung cancer committee. For patients with an enlarged solitary pulmonary nodule that developed after the first operation, CT‐guided or surgical biopsy was performed for tissue diagnosis if indicated, and comprehensive histological subtyping compared with the original tumor to distinguish metachronous from metastatic. Patients with synchronous unresected GGO nodules and metachronous tumors were excluded to distinguish ipsilateral and contralateral recurrence at the beginning of the study.^14^ The disease‐free survival (DFS) was defined as the interval between the date of surgical resection to the date of first recurrence or the last date of follow‐up. An observation was censored at the last follow‐up session at which the patient was alive with recurrence‐free status or had died without recurrence. As of 30 November 2018, all patients had been followed‐up, except for the 69 lost to follow‐up (follow‐up rate 93.5%).

### Statistical analysis

All continuous data are expressed as means and standard deviations. Categorical variables were analyzed by Chi‐square test or Fisher's exact test. Continuous variables were analyzed by the two‐sample *t*‐test. Survival curves were calculated by the Kaplan‐Meier method. A multivariate logistic regression model was used to calculate the score for each patient's propensity for receiving surgeons' preference sublobar resection. Since nonrandom assignment can lead to selection bias and invalidate estimates of survival, we conducted 1‐1 propensity score matching (PSM) based on the following baseline characteristics: age, gender, tumor size, preoperative staging (PET scan), video‐assisted thoracoscopic surgery. Cox regression analysis was then performed on the matched sample for predictors of DFS and OS. Predictors with *P*‐values less than or equal to 0.1 in univariate analyses were included in the multivariate model. Statistical analysis was performed using SPSS Statistics for Windows, Version 22.0 (IBM Corp., Armonk, NY, USA).

## Results

**Figure 2:**
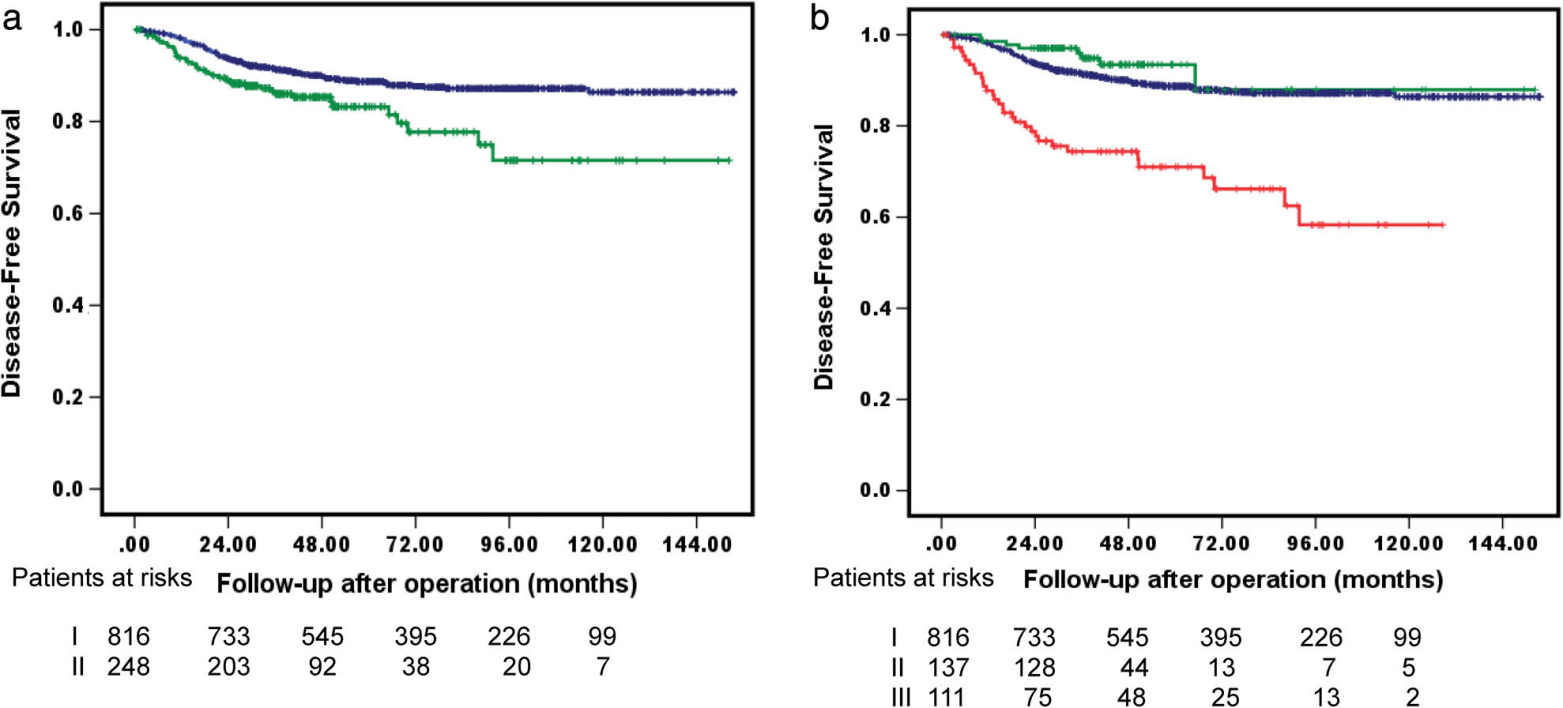
Kaplan‐Meier survival curves for (**a**) DFS in stage I NSCLC <3 cm who underwent lobectomy or sublobar resection, 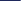 I. Lobectomy, 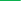 II. Sublobar resection, *p* = 0.002 and (**b**) DFS of patients who underwent lobectomy or medically unfit or surgeons' preference sublobar resections. 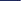 I. Lobectomy, 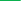 II. Surgeons’ preference sublobar resection, 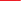 III. Medically unfit, *P <* 0.001.

There was only one postoperative death which occurred at 30 days in this population (0.094%). The median follow‐up period after surgery was 64.5 months, during which the tumor recurred in 132 patients: 55 (41.7%) locoregional‐only recurrences, 31 (23.5%) distal recurrences, and 46 (34.8%) distant with local recurrences. Figure 2a shows the DFS among the patients who underwent lobectomy (*n* = 816) and sublobar resection (*n* = 248); the five‐year DFS was 88.7% vs. 83.2% (*P* = 0.002). Patients who underwent lobectomy, medically unfit (*n* = 111), and surgeons' preference sublobar resection (*n* = 137) had five‐year DFS of 88.7%, 71.0%, and 93.4%, respectively (*P* < 0.001, Fig 2b). To compare the role of surgeons' preference sublobar resection with lobectomy, we further excluded 111 patients with sublobar resection as a compromised procedure (medically unfit, four with central‐type NSCLC), 107 patients with central‐type NSCLC, and 43 patients with limited pulmonary function tests who underwent lobectomy, leaving 803 patients with stage I NSCLC for further analysis. The criteria and demographics of medically unfit patients are provided in Table 1.

**Table 1:** Criteria for medically unfit patients (total 111 patients)

Variable	Number (%)
Preoperative FEV1 or DLCO ≤ 50%	42 (37.8)
Age ≥ 80 years old	46 (41.4)
Age ≥ 75 years old & FEV1 or DLCO ≤ 60%	19 (17.1)
Combined second primary solid malignancy	34 (30.4)
Other comorbidity	11 (9.9)
ESRD under HD	2 (1.8)
Poor LV function: EF ≤ 0.4	3 (2.7)
Recent stroke	3 (2.7)
Intraoperative factors	3 (2.7)

Fit one of above criteria were defined as medically unfit. FEV1 = forced expiratory volume in one second.

DLCO, diffuse capacity of the lung for carbon monoxide; ESRD, end‐stage renal disease; LV, left ventricle.

As shown in Table 2, 666 patients (82.9%) underwent lobectomy (three patients received right upper lobe and right middle lobe bilobectomy for right upper lobe adenocarcinoma, two for incidental right middle lobe PA resection and one for tumor invasion to the right middle lobe) and 137 patients received surgeons' preference sublobar resection (17.1%, including 102 wedge and 35 segmentectomy; without central‐type NSCLC) during this period. The cohort included 350 males (43.6%) and 453 females (56.4%). Mean patient age was 61.5 years (SD = 10.0). Less than one‐third were current or former smokers (27.4%). The mean preoperative serum CEA level was 2.99 ng/mL (SD = 5.29). The preoperative radiological pattern showed 403 (50.2%) patients with solid‐dominant pattern. The mean tumor dimension was 18.1 mm and 312 patients had tumor size more than 2 cm (38.9%). More than half of patients had more than 15 lymph nodes sampling (60.0%) and three mediastinal station lymph nodes sampling (60.8%). The vast majority of tumors were pathologically diagnosed as invasive adenocarcinomas (94.4%). Most patients were at pathological TNM stage pT2a due to pleural invasion. The pleural status of tumor invasion was 447 (55.7%) for PL1 and PL2 combined. Only 199 pathologically‐diagnosed patients had poor differentiation (24.8%) and 79 (9.8%) have angiolymphatic invasion (ALI). Few patients (85, 10.6%) were defined as high grade (micropapillary/solid) predominate pattern by comprehensive histological subtyping. The majority of patients (620, 77.2%) underwent pulmonary resection by video‐assisted thoracic surgery (VATS). In addition, 484 patients (60.3%) had a preoperative whole‐body PET/CT scan to rule out mediastinal and distant metastasis. A total of 27 patients (19.7%, including two patients without records) in the surgeons' preference sublobar resection group were found to have inappropriate margins. Nearly one‐fourth of all patients (181, 22.5%) underwent adjuvant chemotherapy due to a high risk of tumor recurrence. The clinical characteristics of tumor size, radiological appearance, receipt of preoperative PET scan, VATS approach, clinical stage, pathological stage, and quality and quantity of lymph node dissection differed significantly between the lobectomy and surgeons' preference sublobar resection groups (*P* < 0.05).

**Table 2:** Demographics of patient undergoing lobectomy or sublobar resection with surgeons' preference

	Before PSM			After PSM		
	SPSR	Lobectomy		SPSR	Lobectomy	
Variables	(*N* = 137)	(*N* = 666)	*P*‐value	(*N* = 119)	(*N* = 119)	*P*‐value
Pre‐exposure variables						
Age (years old)	61.9 ± 9.6	61.4 ± 10.1	0.621	62.0 ± 9.7	62.9 ± 9.6	0.473
Gender (male)	61 (44.5)	289 (43.4)	0.808	52 (43.7)	52 (43.7)	1.000
Smoking history (yes)	34 (24.8)	186 (27.9)	0.457	30 (25.2)	30 (25.2)	1.000
Preoperative CEA level	3.0 ± 6.3	3.0 ± 5.1	0.994	3.1 ± 6.7	2.9 ± 5.1	0.790
Maximum tumor dimension	1.41 ± 0.54	1.89 ± 0.61	<0.001	1.48 ± 0.54	1.53 ± 0.55	0.520
Radiologic appearance (solid‐dom.)	55 (40.1)	348 (52.3)	0.010	49 (41.2)	53 (44.5)	0.600
Radiologic appearance (pure solid)	29 (21.2)	170 (25.5)	0.282	24 (20.2)	21 (17.6)	0.619
Charlson comorbidity score	2.3 ± 1.3	2.3 ± 1.6	0.996	2.3 ± 1.4	2.4 ± 1.6	0.384
Preoperative PET scan (yes)	55 (40.1)	429 (64.4)	<0.001	55 (46.2)	67 (56.3)	0.120
Surgical method (VATS)	130 (94.9)	490 (73.6)	<0.001	112 (94.1)	117 (98.3)	0.089
Clinical stage			<0.001			0.872
IA1	42 (30.7)	90 (13.5)		31 (26.1)	35 (29.4)	
IA2	81 (59.1)	351 (52.7)		74 (62.2)	70 (58.8)	
IA3	5 (3.6)	135 (20.3)		5 (4.2)	3 (2.5)	
IB	9 (6.6)	90 (13.5)		9 (7.5)	11 (9.2)	
Clinical characteristics						
Margin (inappropriate)	27 (19.7)	‐		25 (21.0)	‐	
Histopathology			0.165			0.389
Invasive adenocarcinoma	128 (93.4)	630 (94.6)		113 (95.0)	114 (95.8)	
Squamous cell carcinoma	0	18 (2.7)		0	3 (2.5)	
Others	9 (6.6)	18 (2.7)		6 (5.0)	2 (1.7)	
Pathological stages			<0.001			0.625
IA1	29 (21.2)	57 (8.6)		19 (16.0)	18 (15.1)	
IA2	39 (28.5)	139 (20.9)		34 (28.6)	33 (27.7)	
IA3	7 (5.1)	85 (12.8)		7 (5.9)	3 (2.5)	
IB	62 (45.2)	385 (57.8)		59 (49.6)	65 (54.6)	
Histology grade (lepidic pred.)	50 (36.5)	111 (16.7)	<0.001	40 (33.6)	31 (26.1)	0.202
Histology grade (high grade pred.)	14 (10.2)	71 (10.7)	0.878	12 (10.1)	13 (10.9)	0.833
Pleural invasion (PL2)	9 (6.6)	73 (11.0)	0.122	8 (6.7)	12 (10.1)	0.350
Histology differentiation (poorly)	32 (23.4)	167 (25.1)	0.672	30 (25.2)	36 (30.3)	0.358
Angiolymphatic invasion (yes)	8 (5.8)	71 (10.7)	0.084	8 (6.7)	11 (9.2)	0.473
Lymph node removed (<15)	97 (70.8)	224 (33.6)	<0.001	79 (66.4)	80 (67.2)	0.891
Lymph node inadequate (station <3)	73 (53.3)	242 (36.3)	<0.001	59 (49.6)	61 (51.2)	0.795
Adjuvant chemotherapy (yes)	23 (16.8)	158 (23.7)	0.077	22 (18.5)	16 (13.4)	0.288

SPSR, surgeons' preference sublobar resection; solid‐dom., solid‐dominant; high‐grade pred., high‐grade predominant; lepidic pred., lepidic predominant.

Table 3 demonstrates the results of univariate and multivariate analyses of DFS for 803 patients who underwent surgical resection. After adjustment of variables in the multivariate model, the surgeons' preference sublobar resection was still not a significant prognostic factor for DFS (adjusted hazard ratio [aHR] = 1.057, 95% confidence interval [CI] = 0.482–2.230, *P* = 0.890). Only radiologic appearance with solid‐dominant tumor (aHR = 2.872, *P* = 0.003), PL2 invasion (aHR = 1.987, *P* = 0.023), or tumor presentation of ALI (aHR = 2.213, *P* = 0.005) were associated with significantly lower rates of DFS.

**Table 3:** Risk analysis of disease‐free survival (803 patients before matching)

	Univariate			Multivariate		
Variables	HR	95% CI	*P*‐value	aHR	95% CI	*P*‐value
Extent of resection (SPSR)	0. 780	0.372–1.637	0.511	1.057	0.482–2.230	0.890
Inappropriate margin of SPSR	2.638	1.061–6.560	0.037	2.007	0.765–5.269	0.157*
Age	1.008	0.984–1.032	0.522	–	–	–
Gender (male)	1.683	1.050–2.698	0.030	1.633	0.920–2.899	0.094
Smoking history (smoker)	1.561	0.957–2.546	0.074	0.839	0.456–1.547	0.575
Preoperative CEA level (ng/ml)	1.026	1.007–1.044	0.006	1.012	0.989–1.036	0.297
Charlson comorbidity score	1.134	0.979–1.314	0.094	1.033	0.881–1.211	0.691
Maximum tumor dimension (cm)	2.361	1.590–3.504	< 0.001	1.593	0.691–3.670	0.275
Radiologic appearance (solid‐dominant)	5.531	2.969–10.306	< 0.001	2.872	1.426–5.782	0.003
Preoperative PET/CT (without)	0.869	0.535–1.411	0.570	–	–	–
Surgical method (VATS)	1.042	0.607–1.788	0.882	–	–	–
Clinical stage	1.629	1.272–2.085	< 0.001	0.858	0.512–1.438	0.561
Clinical characteristics						
Pathological stage	1.414	1.097–1.823	0.008	0.982	0.704–1.368	0.913
Pleural invasion (PL2)	4.036	2.401–6.784	<0.001	1.987	1.098–3.596	0.023
Histology differentiation (poorly)	3.723	2.325–5.963	<0.001	1.570	0.905–2.724	0.108
Angiolymphatic invasion (yes)	4.897	2.955–8.114	<0.001	2.213	1.266–3.870	0.005
Predominate pattern group (High grade)	3.909	2.326–6.570	<0.001	1.716	0.935–3.152	0.081
Lymph node sampling (<15)	1.390	0.867–2.227	0.171	–	–	–
Lymph node station (<3)	0.909	0.559–1.476	0.698	–	–	–
Adjuvant chemotherapy (yes)	1.484	0.890–2.475	0.130	–	–	–

* Adjusted with gender, smoking history, preoperative CEA level, Charlson comorbidity score, maximum tumor dimension, radiologic appearance, stage, pleural invasion (PL2), histology differentiation, angiolymphatic invasion and predominate pattern.

Calculated by Cox regression method; only variables with *P ≤* 0.1 after the univariate analyses were entered into the multivariate model;

SPSR, surgeons' preference sublobar resection.

PSM was performed between lobectomy and surgeons' preference sublobar resection groups, controlling for age, gender, tumor size, preoperative staging (PET scan), and video‐assisted thoracoscopic surgery. The matched sample contained 119 patients who underwent surgeons' preference sublobar resection and 119 patients who underwent lobectomy. No statistical differences were found in any variable between groups after matching (Table 2). The results of risk analysis of DFS in the propensity score‐matched cohort are presented in Table 4. Univariate models revealed that smoking history (hazard ratio [HR] = 3.556, *P* = 0.011), preoperative CEA level (HR = 1.043, *P* = 0.007), radiologic appearance with solid‐dominant tumor (HR = 3.582, *P* = 0.018), clinical stage (HR = 2.143, *P* = 0.005), PL2 invasion (HR = 9.320, *P* < 0.001), poorly differentiated tumor (HR = 5.794, *P* < 0.001), tumor presentation of ALI (HR = 16.236, *P* < 0.001), and high grade predominance of adenocarcinoma (HR = 7.506, *P* < 0.001) significantly increased the risk of DFS. After adjustment of variables in the multivariate model, surgeons' preference sublobar resection remained insignificantly associated with lower rates of DFS (aHR = 0.852, 95% CI = 0.276–2.897, *P* = 0.852). Figure 4 shows the DFS after PSM (*P* = 0.958).

**Table 4:** Risk analysis of disease‐free survival after propensity‐score matching

	Univariate			Multivariate		
Variables	HR	95% CI	*P*‐value	aHR	95% CI	*P*‐value
Extent of resection (SPSR)	1.026	0.383–2.748	0.958	0.852	0.276–2.897	0.852
Inappropriate margin of SPSR	3.736	1.296–10.766	0.015	2.554	0.601–10.854	0.204*
Age	1.035	0.981–1.091	0.211	–	–	–
Gender (male)	2.209	0.737–5.590	0.171	–	–	–
Smoking history (smoker)	3.556	1.331–9.502	0.011	2.127	0.602–7.511	0.241
Preoperative CEA level (abnormal)	1.043	1.011–1.076	0.007	1.003	0.964–1.042	0.898
Charlson comorbidity score	1.275	0.969–1.677	0.082	1.091	0.735–1.619	0.666
Maximum tumor dimension	2.408	0.996–5.825	0.051	0.234	0.021–2.620	0.239
Radiologic appearance (solid‐dominant)	3.582	1.239–10.354	0.018	2.125	0.536–8.423	0.283
Preoperative PET/CT (without)	1.172	0.435–3.160	0.753	–	–	–
Surgical method (VATS)	0.821	0.107–6.295	0.849	–	–	–
Clinical stage	2.143	1.261–2.143	0.005	2.213	0.555–8.823	0.260
Clinical characteristics						
Pathological stage	1.299	0.842–2.004	0.237	–	–	–
Pleural invasion (PL2)	9.320	3.333–26.067	<0.001	1.916	0.422–8.710	0.400
Histology differentiation (poorly)	5.794	2.089–16.066	0.001	1.504	0.299–7.572	0.621
Angiolymphatic invasion (yes)	16.236	5.878–44.849	<0.001	7.114	1.295–39.076	0.024
Predominate pattern group (high grade)	7.506	2.781–20.254	<0.001	1.130	0.219–5.832	0.884
Lymph node sampling (<3)	2.901	0.656–12.821	0.160	–	–	–
Lymph node station (<15)	1.383	0.499–3.833	0.533	–	–	–
Adjuvant chemotherapy (yes)	1.867	0.602–5.793	0.280	–	–	–

* Adjusted with smoking history, preoperative CEA level, Charlson comorbidity score, maximum tumor dimension, radiologic appearance, pleural invasion (PL2), histology differentiation, angiolymphatic invasion and predominate pattern.

Calculated by Cox regression method; only variables with *P ≤* 0.1 after the univariate analyses were entered into the multivariate model;

SPSR, surgeons' preference sublobar resection.

## Discussion

The Lung Cancer Study Group published the results of the only randomized study comparing lobectomy with sublobar resection for stage I lung cancer in 1995. Higher death rates and threefold‐higher local recurrence rates were reported in patients who underwent sublobar resection.^15^ Based on these results, a lobectomy was considered the procedure of choice for patients with early‐stage lung cancer. In recent years, studies with strict and well‐defined patient selection criteria have demonstrated comparable oncological outcomes in selected patients for sublobar resection versus lobectomy for early stage NSCLC, yet others have not.^1^, ^2^, ^16^, ^17^

The present study retrospectively compared oncological outcomes between patients who underwent lobectomy and intentional sublobar resection as a parenchyma‐sparing procedure in patients who could tolerate lobectomy for stage I NSCLC <3 cm. In the initial overall cohort, DFS was significantly worse in the sublobar resection group (Fig 2a, *P* = 0.002). However, the survival curves depicted a significant separation between those receiving sublobar resection and those who had “compromised” purposes (medically unfit) or surgeons' preference sublobar resection (Fig 2b, *P* < 0.001). To date, however, no consensus has been reached on the definition of “medically unfit” or “compromised” sublobar resection.^18^ In the present study, the reasons for compromised‐purpose sublobar resection were recorded in the operative notes according to the individual thoracic surgeon's preference; these reasons were similar to the majority of reasons for high‐risk patients undergoing lobectomy as reported in the literature.^3^ The present study also demonstrated that this group of high‐risk patients as adjudged by thoracic surgeons had a significantly worse DFS compared to the patients without high risk who underwent either lobectomy or surgeons' preference sublobar resection (Fig 2b).

Heterogeneity of the population studied is one possible explanation for the controversy surrounding the extent of lung resection, even in patients with stage I NSCLC. For example, some studies found that established factors favored sublobar resection.^19^, ^20^ Many studies have demonstrated excellent surgical outcomes for really “early” stage lung cancers, pre‐ or minimally invasive adenocarcinoma.^21^, ^22^ However, some studies found that lobectomy remained the standard therapy for small (≤20 mm) NSCLC.^23^, ^24^ Both types of studies excluded those with “bronchoalveolar carcinoma” from their cohorts. Similarly, we excluded patients with pre‐ or minimally invasive adenocarcinoma as proposed by the IASLC, American Thoracic Society, and European Respiratory Society.^25^ In the present study, the oncological outcomes of surgeons' preference sublobar resection for stage I NSCLC <3 cm, without pre‐ or minimally invasive adenocarcinoma, remained comparable with the outcomes of lobectomy.

**Figure 3:**
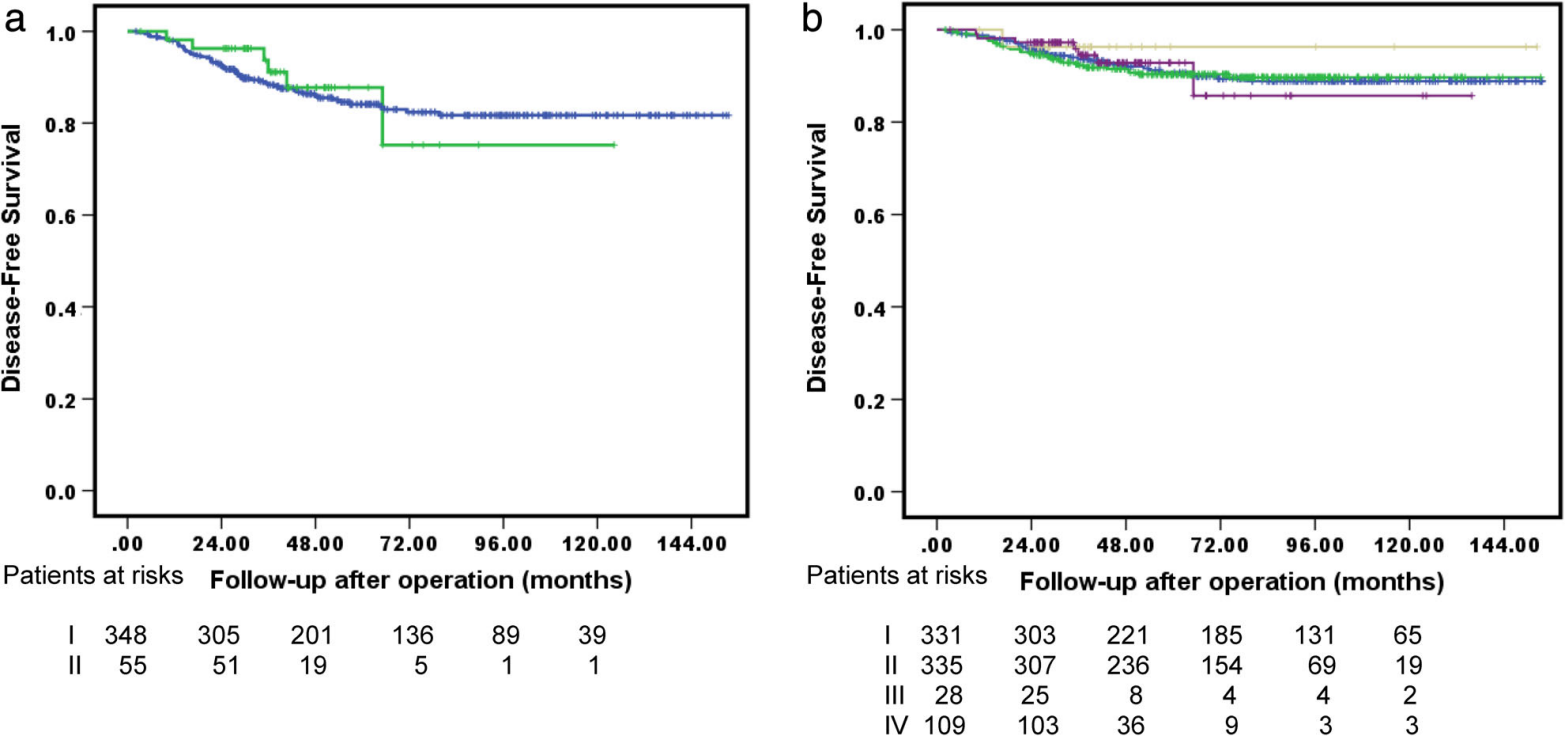
Kaplan‐Meier survival curves for (**a**) DFS in subgroup analysis with solid‐dominant stage I NSCLC <3 cm who underwent lobectomy or sublobar resection 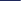 I. Lobectomy, 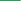 II. Surgeons’ preference sublobar resection, *P* = 0.721 and (**b**) DFS of patients subgroup who underwent extent of resection combined with lymphadenectomy. 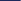 I. Lobectomy + LN (+), 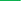 II. Lobectomy + LN (−), 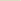 III. Surgeons’ preference sublobar resection + LN (+), 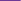 IV. Surgeons’ preference sublobar resection + LN (−), *P =* 0.885.

Another issue driving the extent of resection is the aggressiveness of these peripheral small lung cancers. A crucial clinical factor in tumor aggressiveness is the radiographic appearance of the tumor: CTR.^26^ Nishio *et al*. reported that, in comparison of 59 CTR >0.5 patients with propensity score‐matched pairs, the five‐year local‐regional recurrence‐free survival rate for those receiving segmentectomy was 76.3% versus 91.5% for lobectomy (*P* = 0.082). Multivariate analysis further confirmed that segmentectomy was the only independent risk factor associated with local‐regional recurrence‐free survival (*P* = 0.020).^27^ However, Altorki *et al*. and Koike *et al*. reported equivalent survival for sublobar resection or lobectomy in patients with clinical stage IA lung cancer with radiological solid nodules.^2^, ^28^ In the present study, the solid‐dominant tumor was significantly associated with an increased risk of DFS in all cohorts (aHR = 2.908, *P* = 0.003), except the PSM cohort (aHR = 1.374, *P* = 0.636). As shown in Fig 3a, subgroup analysis in all 403 solid‐dominant patients demonstrated an equivalent DFS for surgeons' preference sublobar resection and lobectomy (five‐year DFS 87.7%, and 84.1%, respectively, *P* = 0.721).

**Figure 4:**
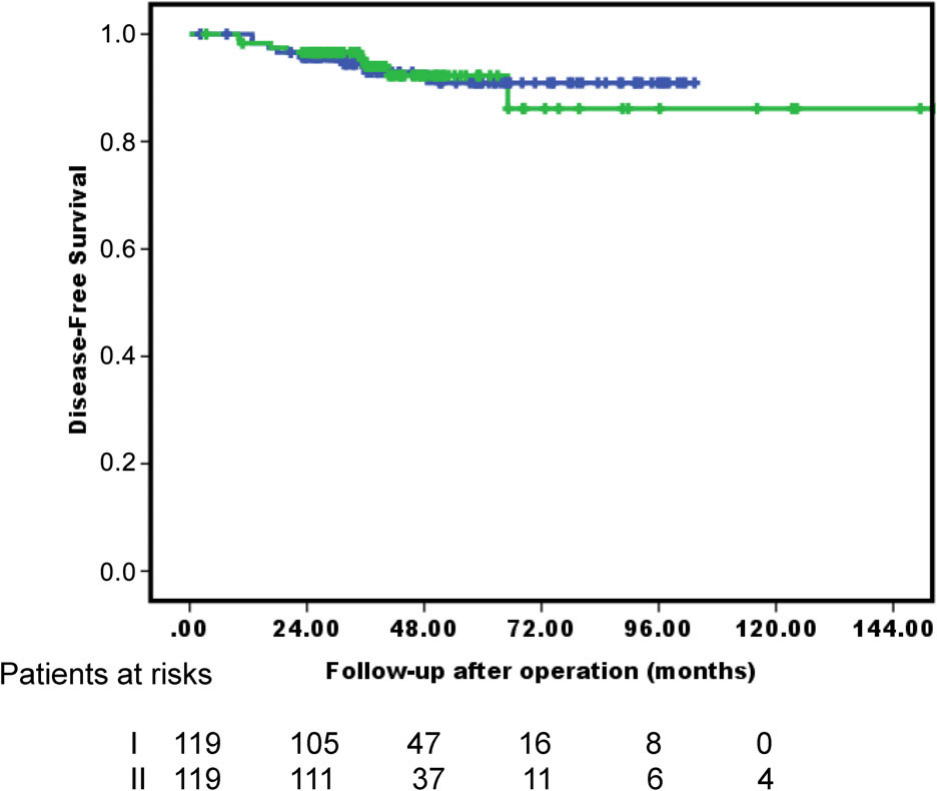
Kaplan‐Meier survival curves for DFS of patients who underwent lobectomy or received surgeons' preference sublobar resection after PSM. 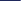 I. Lobectomy, 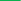 II. Surgeons’ preference sublobar resection, *P* = 0.958.

The other clinical surrogate of tumor aggressiveness is the tumor maximum standard uptake value (SUVmax) from the preoperative PET/CT. Those cases with higher SUVmax are usually excluded from consideration for sublobar resection at the surgeon's discretion, in response to research correlating SUVmax and pathological findings.^29^ Kamel *et al*. reported that lobectomy and segmentectomy are comparable oncologic procedures for patients with carefully staged cT1 N0 lung adenocarcinoma with hypermetabolic tumors (SUVmax ≥3 g/dL).^30^ In the present study, only eight patients with PET SUVmax >3 underwent surgeons' preference sublobar resection due to surgeons' discretion, a case number too small to enable conclusions to be made. Additionally, the PET SUVmax might not be consistently measured between facilities, hindering further comparison.

The other issue regarding sublobar resection is that it is usually done in combination with inadequate lymph node sampling,^16^, ^17^, ^30^ in particular for N1 segmental lymph nodes. Sublobar resection and inadequate lymph node sampling are endorsed as quality metrics for surgical resection to treat stage IB NSCLC according to the Lung Cancer Treatment Quality Control Program in Taiwan and other clinical guidelines.^7^, ^31^ In addition, unexpected N1 or N2 disease ranges 4%–7%, in the experience of Altorki and colleagues,^2^ a report in line with the most recent literature. Similar to previous studies,^16^, ^17^ patients in the present study who underwent further sublobar resection had inadequate lymphadenectomy (either quality or quantity), but neither issue was significantly associated with DFS in the total cohort or PSM analyses. As shown in Fig 3b, subgroup analysis of the extent of resection and adequate lymphadenectomy demonstrated an equivalent five‐year DFS between the stratified groups: lobectomy with adequate lymphadenectomy, lobectomy with inadequate lymphadenectomy, surgeons' preference sublobar resection with adequate lymphadenectomy, and surgeons' preference sublobar resection with inadequate lymphadenectomy (90.7%, 90.3%, 96.3%, and 92.8%, respectively, *P* = 0.885). Although lobectomy was associated with more thorough lymph node sampling, this did not translate into a higher rate of DFS compared with the DFS rates of sublobar resection.^30^ Similar to the previous ACOSOG Z0030 randomized trial, the present study failed to show differences in recurrence or survival advantage between adequate and inadequate lymph node sampling.

Most previous studies that found favorable outcomes with sublobar resection indicated that this procedure was for T1N0M0 NSCLC of 2 cm or less.^2^, ^19^, ^20^ The present study included 312 patients with a whole tumor size of 2–3 cm (38.9%). Similar to the results of previous studies, patients with T1b lung adenocarcinoma selected on the basis of radiological pattern and PET/CT findings were potential candidates for sublobar resection with a sufficient surgical margin.^1^, ^32^ In the present study, tumor size was not significantly associated with an increased risk of DFS in all cohorts (aHR = 1.338, *P* = 0.267). In addition, subgroup analysis in all 312 tumors sized 2–3 cm demonstrated an equivalent five‐year DFS for surgeons' preference sublobar resection and lobectomy (76.2% and 86.5%, respectively, *P* = 0.279). Therefore, a future prospective, randomized study is needed to compare sublobar resection and lobectomy for T1N0M0 NSCLC sized 2.1–3 cm. Margins are one of the well known problems associated with sublobar resections. Otherwise, in the present study, inappropriate margins were not significantly associated with increased risk of DFS in all cohorts (aHR = 2.004, *P* = 0.159), nor in the PSM cohort (aHR = 2.095, *P* = 0.275), after adjusting for other significant factors (Tables 3, 4).

This study has several limitations. Because the analysis was retrospective, the patient population undergoing intentional sublobar resection may have been highly selective after excluding the compromised procedures from the operative reports. This also may reflect a possible bias of different surgeons, which has not been validated elsewhere. Although PSM was used to compare lobectomy and surgeons' preference sublobar resection in the present study, the cohort represented similar clinical and pathological characteristics between the two groups, consequently resulting in comparable surgical outcomes. However, PSM did not represent a comparable oncological outcome between sublobar resection and lobectomy in the borderline unbiased patients with stage I NSCLC. Traditionally, anatomic segmentectomy has been considered superior to wedge resection. In the present study, the majority of surgeons' preference sublobar resections were performed as wedge resections, not segmentectomy. The subgroup analysis in all 137 surgeons' preference sublobar resection patients demonstrated an equivalent DSF for segmentectomy and wedge resection (five‐year DFS 92.9%, and 93.0%, respectively, *P* = 0.761). Results were similar to those of several studies comparing different types of sublobar procedures performed for early‐stage lung cancer that failed to show a survival benefit of one procedure over the other.^1^, ^32^ In addition, total tumor size, rather than radiological solid tumor size or pathological invasive size, was recorded and analyzed in this study and we neither performed analysis of the microscopic spread through the alveolar space (STAS), or considered risk factors for tumor recurrence after sublobar resection. However, results of the present study provide an important reflection of “real world” outcomes for patients who undergo sublobar resection in peripheral early stage lung cancer.

In conclusion, surgeons' preference sublobar resection is not an independent predictor of DFS. Carefully selected patients who undergo surgeons' preference sublobar resection have comparable outcomes to those receiving lobectomy for stage I NSCLC <3 cm. Large randomized trials are underway to define the clinical role of sublobar resections, and results are eagerly anticipated. Until that time, lobectomy should still be regarded as the mainstay of surgical therapy, especially for patients with known high‐risk of recurrent predictors such as radiographic solid‐appearance and tumors with high SUVmax uptake. Apart from that, sublobar resection may be considered as a useful treatment alternative for select stage I NSCLC patients.

## Disclosure

The authors declare that there are no potential conflicts.
